# Identification of key genes associated with cervical cancer based on bioinformatics analysis

**DOI:** 10.1186/s12885-024-12658-z

**Published:** 2024-07-25

**Authors:** Xinmeng Yang, Mengsi Zhou, Yingying Luan, Kanghua Li, Yafen Wang, Xiaofeng Yang

**Affiliations:** 1 Department of Obstetrics and Gynecology, Zhengzhou Central Hospital Affiliated to Zhengzhou University, Zhengzhou, 450007 China; 2 Department of Reproductive Medicine, The Third Affiliated Hospital of Zhengzhou University, Zhengzhou, 450015 China; 3 Laboratory Department, Zhecheng County People’s Hospital, Shangqiu, 476299 China

**Keywords:** Cervical cancer, Differentially expressed genes, Bioinformatics analysis, Hub genes

## Abstract

**Background:**

Cervical cancer has extremely high morbidity and mortality, and its pathogenesis is still in the exploratory stage. This study aimed to screen and identify differentially expressed genes (DEGs) related to cervical cancer through bioinformatics analysis.

**Methods:**

GSE63514 and GSE67522 were selected from the GEO database to screen DEGs. Then GO and KEGG analysis were performed on DEGs. PPI network of DEGs was constructed through STRING website, and the hub genes were found through 12 algorithms of Cytoscape software. Meanwhile, GSE30656 was selected from the GEO database to screen DEMs. Target genes of DEMs were screened through TagetScan, miRTarBase and miRDB. Next, the hub genes screened from DEGs were merged with the target genes screened from DEMs. Finally, ROC curve and nomogram analysis were performed to assess the predictive capabilities of the hub genes. The expression of these hub genes were verified through TCGA, GEPIA, qRT-PCR, and immunohistochemistry.

**Results:**

Six hub genes, *TOP2A*, *AURKA*, *CCNA*2, *IV*L, *KRT1*, and *IGFBP*5, were mined through the protein-protein interaction network. The expression of these hub genes were verified through TCGA, GEPIA, qRT-PCR, and immunohistochemistry, and it was found that *TOP2A*, *AURKA* as well as *CCNA*2 were overexpressed and *IGFBP5* was low expression in cervical cancer.

**Conclusions:**

This study showed that *TOP2A*, *AURKA*, *CCNA*2 and *IGFBP5* screened through bioinformatics analysis were significantly differentially expressed in cervical cancer samples compared with normal samples, which might be biomarkers of cervical cancer.

**Supplementary Information:**

The online version contains supplementary material available at 10.1186/s12885-024-12658-z.

## Background

Cervical cancer (CC) is the fourth most common cancer and the fourth leading cause of death from cancer in women worldwide in 2020. According to statistics, it was estimated that there were 604,127 new cases and 341,831 deaths worldwide in 2020, accounting for 6.5% of new female cancer cases and 7.7% of deaths [1, 2]. The most common types of cervical cancer are squamous cell carcinoma (SCC) and adenocarcinoma (ADC), accounting for 70% and 25%, respectively. And a less common type of cervical cancer is adenosquamous carcinoma (ADSC) [3, 4]. Acknowledgedly, HPV is an important factor that causes cervical cancer [5]. Due to cytological screening, HPV vaccination and other methods, the incidence of cervical cancer is greatly reduced [3, 6]. The treatment methods of cervical cancer include surgery, chemotherapy and radiotherapy [7]. Although the level of treatment has improved in recent years, the long-term prognosis of cervical cancer is still poor because of drug resistance and recurrence.

Most patients with localized cervical cancer can be cured by surgery, and the 5-year survival rate is 91.5%. However, patients with metastatic cervical cancer still have no good treatment methods, and the 5-year survival rate is low, only 17% [4, 8]. In recent years, with the development of high-throughput sequencing technology and bioinformatics analysis methods, the role of differentially expressed genes (DEGs) in cervical cancer has been continuously explored. Therefore, mining DEGs from the molecular level has become a necessary choice to provide new ideas for the diagnosis and prognosis of cervical cancer.

In this study, two microarray datasets (GSE63514 and GSE67522) were downloaded from the GEO database using bioinformatics methods to screen DEGs. The DEGs were performed to Gene Ontology (GO) analysis and Kyoto Encyclopedia of Genes and Genomes ( KEGG) pathway enrichment analysis. And then a protein-protein interaction (PPI) network was constructed to screen hub genes. The GSE30656 dataset was downloaded from the GEO database to screen differentially expressed miRNAs (DEMs). The target genes of DEMs were predicted through the three websites of Targetscan, miRDB and miRTarBase. The final DEGs were screened by the intersection of the hub genes and the target genes. The Cancer Genome Atlas (TCGA) and Gene Expression Profile Interaction Analysis (GEPIA) database were used to identified these gene screened. RT-qPCR and immunohistochemistry were used to verify the results.This study provided new perspectives and ideas for further research on the mechanism of cervical cancer occurrence and development.

## Materials and methods

### Data collection

**Fig. 1:**
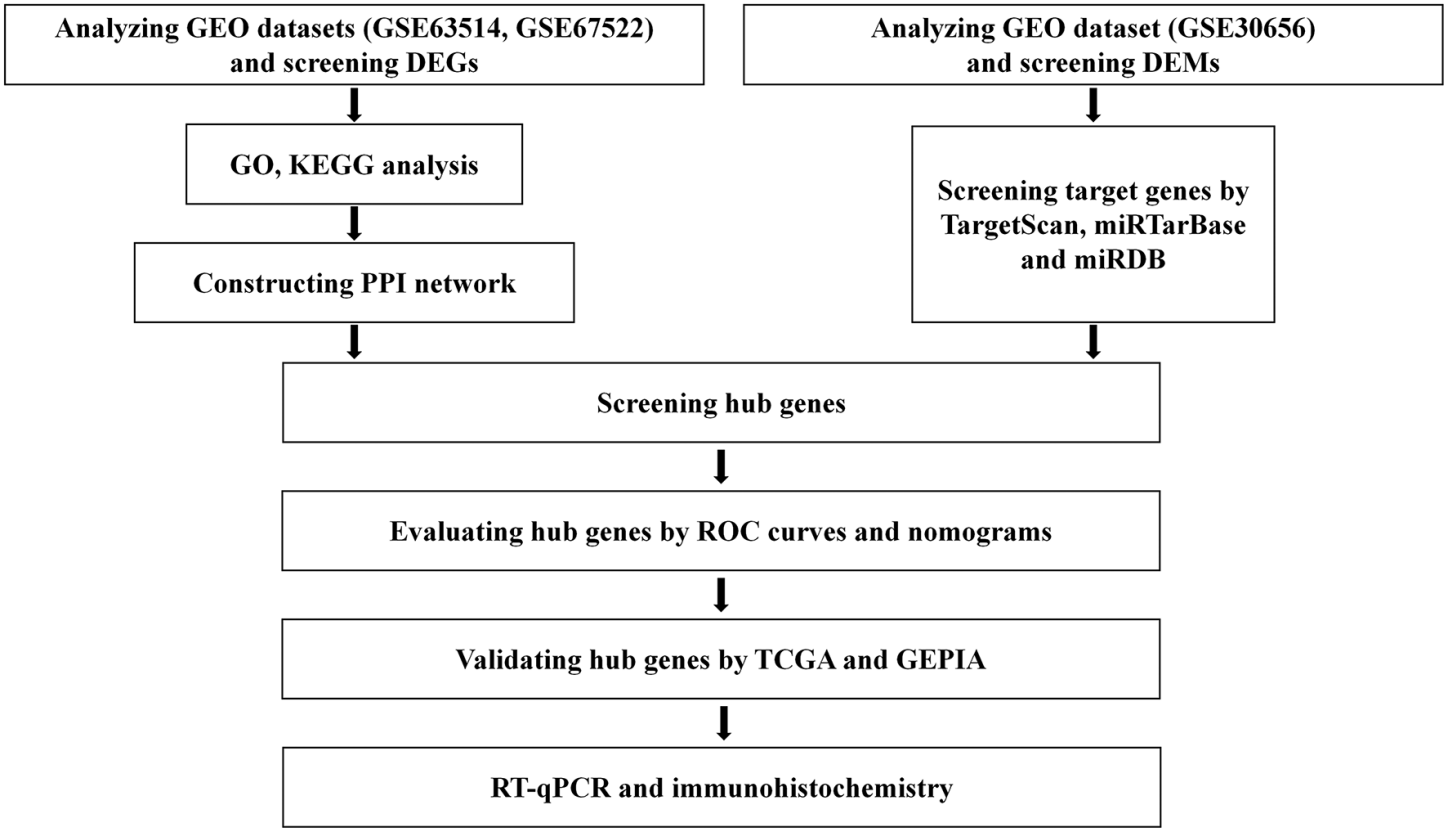
The flowchart of the methodology

Figure 1 depicted the study fowchart.

The cervical cancer datasets were download from the GEO database (https://www.ncbi.nlm.nih.gov/geo/). The search terms included “cervical cancer”, “homo sapiens”, “expression profiling by array”, and we chose the datasets including normal tissues and tumor tissues. GSE63514 is a gene expression analysis of cervical cancer progression on 24 normal specimens and 28 cancer specimens. GSE67522 includes 22 normal and 28 cancers specimens to identify the probably functionally relevant pathways in cervical cancer progression. To exclude the effect of individual heterogeneity and ensure more accurate results, we chose these two datasets for further analysis. Then we searched for “cervical cancer”, “homo sapiens”, “Non-coding RNA profiling by array”, and finally GSE30656 dataset was selected for analysis. GSE30656 includes 10 squamous cell carcinomas of the cervix, 9 adenocarcinomas of the cervix, and 10 cervical squamous epithelial samples with normal histology, which were used to identify miRNAs associated with cervical carcinogenesis.

### Screening of DEGs and DEMs

GEO2R (https://www.ncbi.nlm.nih.gov/geo/info/geo2r.html) performed comparisons on original submitter-supplied processed data tables using the GEOquery and limma R packages from the Bioconductor project. Two mRNA datasets (GSE63514 and GSE67522) and miRNA datasets (GSE30656) were analyzed using GEO2R. We defined DEGs that met the two screening conditions of adjusted (adj.) p value < 0.05 as well as |log_2_ fold change (log_2_FC)| > 1.5 were statistically significant. The DEGs were analyzed with Venny 2.1.0 (http://bioinfogp.cnb.csic.es/tools/venny/index.html), and then the common up-regulated and down-regulated DEGs were obtained. The miRNAs with the largest difference multiple were selected as the object of follow-up study.

### Functional enrichment analysis of DEGs

GO enrichment analysis and KEGG signaling pathway analysis were performed on the above DEGs by DAVID 6.7 (https://david-d.ncifcrf.gov/) to understand the biological functions of these DEGs [9]. Fill in gene ID in “Enter Gene List”, select “OFFICIAL_GENE_SYMBOL” in “select identifier”, select “Homo sapiens” in “Select species”. Finally, the KEGG results were visualized by R language. Corrected P-Value < 0.05 indicated statistical difference.

### PPI network construction

Protein-protein interaction analysis can serve as an entry point to better explain the relationships between different proteins at the genome scale, and may help provide new insights into the functional interpretation of proteins. STRING (version 11.0, https://string-db.org/) database, which has 5090 organisms and 24.6 million proteins, was used to construct the PPI network [10]. “Network type” was set to “full STRiNG network (the edges indicate both functional and physical protein associations)”. “Meaning of network edges” was set to “evidence”. “Minimum required interaction score” was set to “medium confidence (0.400)”. “Max number of interactors to show” was set to “none”. The analyzed data was imported into Cytoscape (version 3.6.1, http://www.cytoscape.org/) software, and the top 10 genes were selected through 12 algorithms [11], and then the hub genes with the most frequency were screened. The hub genes with the highest score were selected according to Degree score. These screened hub genes were used as research objects.

### Screening of DEMs target genes

TargetScan (http://www.targetscan.org/), miRTarBase (http://mirtarbase.cuhk.edu.cn/php/index.php) and miRDB (http://mirdb.org/) were applied to predict the potential target genes of DEMs. The target genes of DEMs were screened using Venny online software. Conditions for screening target genes of TargetScan: Select “Human” in “Select a species”, and fill in “miRNA” in “Enter a microRNA name”. Conditions for screening target genes of miRTarBase: Select “Human” in “Species”, and then click “Submit”. Conditions for screening target genes of miRDB: Select “Human” in “Search by miRNA name”, and then fill in the miRNAs to be searched.

### ROC curve and nomogram analysis

The receiver operating characteristic (ROC) curve was a simple, efficient and comprehensive tool. It constructed a monotonically increasing curve by connecting the values of the true positive rate and the false positive rate at different cutoff points or thresholds. The area under the curve (AUC) could be used as an indicator to measure the diagnostic effect. The larger the area, the more effective the classification method was. Nomogram analysis transformed the complex regression equation into a visual graph, and making the results of the prediction model more readable. ROC curve was drawn by GraphPad Prism 9.0.0, and the nomogram was drawn by the Hmisc and rms packages of R language.

### Validation of hub genes and DEMs

TCGA (The Cancer Genome Atlas) is a large-scale cancer research project jointly established by the National Cancer Institute (NCI) and the National Human Genome Research Institute (NHGRI). It molecularly characterized over 20,000 primary cancer and matched normal samples spanning 33 cancer types, which is a free and open cancer genetic research database. GEPIA (http://gepia.cancer-pku.cn/) is a cancer data analysis website developed by the Peking University team, which is based on TCGA and Genotype-Tissue Expression (GTEx) database.

The TCGA database and GEPIA were used to verify the expression of hub genes in cervical cancer samples, and RT-qPCR technology was used to verify the relative mRNA expression levels of hub genes and miRNA expression levels of DEMs in cervical cancer cells.

### Cell lines and cell culture

Ect1/E6E7 cell (Human cervix immortalized squamous cells) as well as Hela and SiHa cell (Human cervical cancer cell lines) were obtained from Chinese Academy of Sciences Cell Bank/Stem Cell Bank (Shanghai, China).These cell lines were cultured in DMEM medium supplemented with 10% fetal bovine serum and 1% penicillin and streptomycin at 37˚C in a humidified atmosphere of 5% CO2. 500,000 Ect1/E6E7 and Hela as well as SiHa cells were respectively seeded in the wells of the six-well plate for RNA extraction, and the experiment was repeated three times.

### RNA extraction and RT-qPCR

miRNA extraction and RT-qPCR: Total RNA was extracted from the cultured cells using Trizol Reagent (Solaibao Technology Co. LTD, Beijing, China) following the manufacturer’s protocol. For the detection of miRNA expression, complementary DNA (cDNA) was synthesized with miRNA 1st Strand cDNA Synthesis Kit by stem-loop (Vazyme Biotech Co.,Ltd, Nanjing, China) and qPCR was performed with miRNA Universal SYBR^®^ qPCR Master Mix (Vazyme Biotech Co.,Ltd). The relative expression level of miRNA was normalized to U6 small nuclear RNA (U6).

mRNA extraction and RT-qPCR: To analyze mRNA expression levels, total RNA was reversed transcribed to cDNA using FastKing RT Kit with gDNase (Tiangen Biochemical Technology Co., LTD, Beijing, China) and qPCR was performed with Hieff^®^ qPCR SYBR Green Master Mix (Low Rox Plus). The relative expression level of mRNA were normalized to β-ACTIN.

The operating conditions used for qPCR of miRNA and mRNA were as follows: hold stage was 95˚C for 5 min, PCR stage were the 40 cycles of 95˚C for 10 s and 60˚C for 30 s, and the melt curve stage were 95˚C for 15 s and 60˚C for 1 min as well as 95˚C for 15 s. All experiments were performed in triplicate. Data were analyzed using the comparative Ct (2-ΔΔCt) method for quantification. The primer sequences were shown in Table 1.

**Table 1:** The primer sequences

Gene	The forward primer	The reverse primer
TOP2A	AAGATTCATTGAAGACGCTTCG	GCTGTAAAATGCCATTTCTTGC
IGFBP5	ACCCAGTCCAAGTTTGTCGG	AATTGGGCAGGTACACAGCA
KRT1	CCGAAGGAGAGTGGACCAAC	CTCTGCATTTGTCCGCTTGT
AURKA	CTTCCCAGCGCATTCCTTTG	TGAGGTACACTGGTTGCCTG
IVL	GCTCCTCAAGACTGTTCCTCC	CAGGCAGTCCCTTTACAGCA
β-ACTIN	CCTGGCACCCAGCACAAT	GGGCCGGACTCGTCATAC
miRNA-21-3P	ACCGAGGTCAACACCAGTCGA	AGTGCAGGGTCCGAGGTATT
miRNA-21-5P	CGCCGTAGCTTATCAGACTGA	AGTGCAGGGTCCGAGGTATT
miRNA-203a-3P	CCGCGTGAAATGTTTAGGACC	AGTGCAGGGTCCGAGGTATT
miRNA-203a-5P	GCACGTCCAGTGGTTCTTAACAG	AGTGCAGGGTCCGAGGTATT
miRNA-203b-3P	CCGCCTTGAACTGTTAAGAACCA	AGTGCAGGGTCCGAGGTATT
miRNA-203b-5P	GAGCGCGTAGTGGTCCTAAACA	AGTGCAGGGTCCGAGGTATT
U6	CTCGCTTCGGCAGCACA	AACGCTTCACGAATTTGCGT
miRNA-21-3P-loop	GTCGTATCCAGTGCAGGGTCCGAGGTATTCGCACTGGATACGACACAGCC	
miRNA-21-5P-loop	GTCGTATCCAGTGCAGGGTCCGAGGTATTCGCACTGGATACGACTCAACA	
miRNA-203a-3P-loop	GTCGTATCCAGTGCAGGGTCCGAGGTATTCGCACTGGATACGACCTAGTG	
miRNA-203a-5P-loop	GTCGTATCCAGTGCAGGGTCCGAGGTATTCGCACTGGATACGACAACTGT	
miRNA-203b-3P-loop	GTCGTATCCAGTGCAGGGTCCGAGGTATTCGCACTGGATACGACTCCAGT	
miRNA-203b-5P-loop	GTCGTATCCAGTGCAGGGTCCGAGGTATTCGCACTGGATACGACTGTGAA	

### Immunohistochemical analysis

The protein expression of hub gene in cervical cancer tissue was analyzed through the Human Protein Atlas database (http://www.proteinatlas.org). According to the staining intensity of protein in the tissue and percentage of stained cells, compared the protein expression of the DEGs in normal and tumor tissue, and captured representative immunostaining images.

## Results

### Screening for differentially expressed genes

**Fig. 2:**
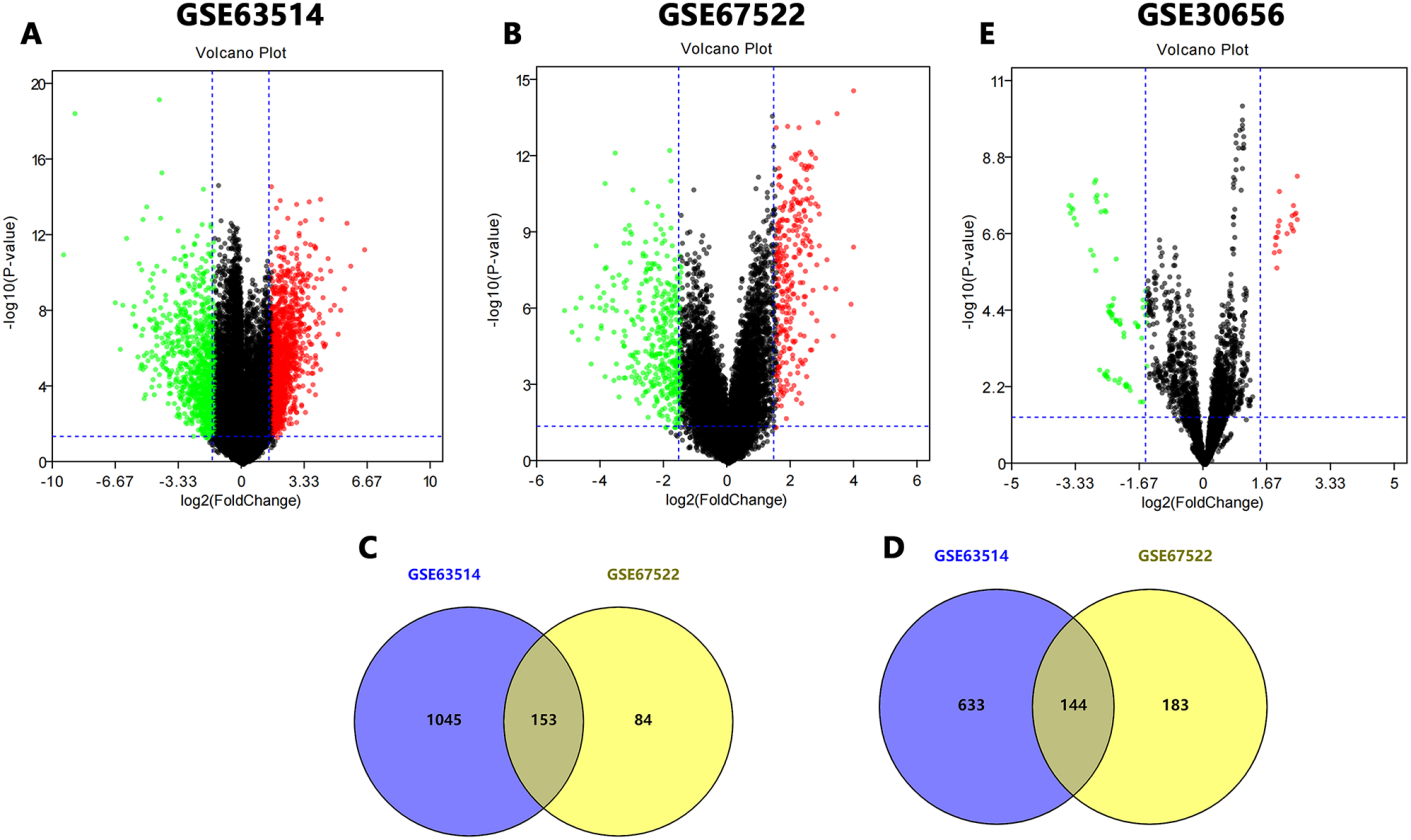
The volcano plots and venn diagrams of DEGs and DEMs. (**A**) GSE63514 volcano plot. (**B**) GSE67522 volcano plot. (**C**) 153 up-regulated DEGs shared by two GEO datasets. (**D**) 144 down-regulated DEGs shared by two GEO datasets. (**E**) GSE30656 volcano plot

Cervical cancer and normal cervical epithelial tissues were compared in the GSE63514 and GSE67522 dataset by GEO2R. There were 1975 DEGs in GSE63514, including 1198 up-regulated genes and 777 down-regulated genes. GSE67522 screened 564 DEGs with 237 up-regulated genes and 327 down-regulated genes. The volcano plots of these DEGs for each dataset were shown in Fig. 2A-B. The above DEGs were analyzed by Venny 2.1.0 and it was found that there were 153 co-upregulated genes and 144 co-downregulated genes, as shown in C-D of Fig. 2. Detailed results were shown in Table S1. GEO2R was used to analyze the GSE30656 dataset, and the volcano plot of DEMs was shown in Fig. 2E. miR-21 and miR-203 with the largest difference multiple were selected as subsequent research objects. The information of the three datasets were shown in Table 2.

**Table 2:** GEO data information

Dataset	Platform	Year	Tumor Tissues	Normal Tissues	Total Tissues	Up-regulated genes	Down-regulated genes	Total genes
GSE63514	GPL570	2015	28	24	52	1198	777	1975
GSE67522	GPL10558	2015	28	22	50	237	327	564
GSE30656	GPL6955	2012	19	10	29	1	5	6

### GO functional annotation and KEGG enrichment analysis of DEGs

**Fig. 3:**
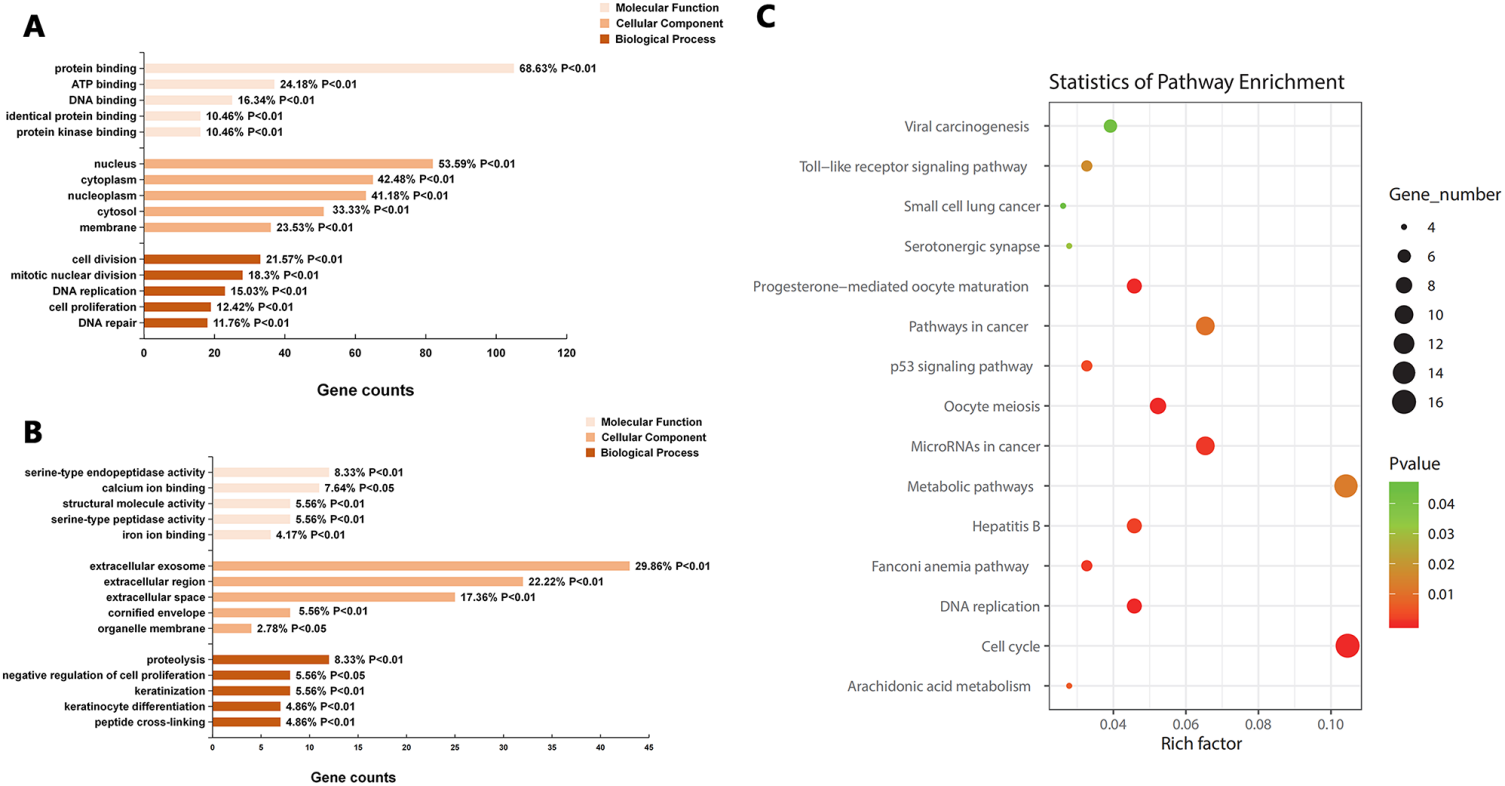
GO functional annotation and KEGG enrichment analysis of the DEGs. (**A**) GO functional annotation of up-regulated DEGs. (**B**) GO functional annotation of down-regulated DEGs. (**C**) KEGG pathway enrichment analysis of DEGs

The GO functional annotation of the screened DEGs were obtained by using the DAVID database including the following three parts: molecular function (MF), cellular component (CC) and biological process (BP). The top 15 up-regulated GO terms and down-regulated GO terms were summarized in Table 3. As shown in Fig. 3A, up-regulated DEGs were mainly enriched in protein binding (MF), nucleus (CC), and cell division (BP). The down-regulated DEGs were mostly concentrated in serine-type endopeptidase activity (MF), extracellular exosome (CC) and proteolysis (BP), as shown in Fig. 3B. The KOBAS online analysis tool was used to analyze the KEGG pathway of DEGs, and the bubble map was drawn using R language, as shown in Fig. 3C. The KEGG pathway of DEGs were mainly enriched in Cell cycle and Metabolic pathways, and the enrichment pathway of DEGs were shown in Table 4.

**Table 3:** Enriched Gene Ontology terms of the upregulated and downregulated DEGs

Category	Term	Count	P-value	State
BP	DNA repair	18	1.81959E-11	Upregulated
BP	cell proliferation	19	2.43866E-09	Upregulated
BP	DNA replication	23	6.8736E-21	Upregulated
BP	mitotic nuclear division	28	2.37475E-22	Upregulated
BP	cell division	33	4.77225E-24	Upregulated
CC	membrane	36	3.99517E-05	Upregulated
CC	cytosol	51	1.98869E-06	Upregulated
CC	nucleoplasm	63	1.59763E-15	Upregulated
CC	cytoplasm	65	4.82211E-05	Upregulated
CC	nucleus	82	4.33561E-11	Upregulated
MF	protein kinase binding	16	5.44987E-07	Upregulated
MF	identical protein binding	16	0.001398503	Upregulated
MF	DNA binding	25	0.005436523	Upregulated
MF	ATP binding	37	3.21277E-09	Upregulated
MF	protein binding	105	5.04978E-08	Upregulated
BP	peptide cross-linking	7	1.66823E-06	Downregulated
BP	keratinocyte differentiation	7	1.96534E-05	Downregulated
BP	keratinization	8	5.46788E-08	Downregulated
BP	negative regulation of cell proliferation	8	0.026580792	Downregulated
BP	proteolysis	12	0.001119847	Downregulated
CC	organelle membrane	4	0.026836896	Downregulated
CC	cornified envelope	8	4.40417E-08	Downregulated
CC	extracellular space	25	4.89321E-05	Downregulated
CC	extracellular region	32	5.80683E-07	Downregulated
CC	extracellular exosome	43	3.38145E-06	Downregulated
MF	iron ion binding	6	0.005623021	Downregulated
MF	serine-type peptidase activity	8	4.0432E-07	Downregulated
MF	structural molecule activity	8	0.002435629	Downregulated
MF	calcium ion binding	11	0.040259422	Downregulated
MF	serine-type endopeptidase activity	12	3.06287E-06	Downregulated

**Table 4:** The enriched pathways of DEGs

ID	Pathway	Gene number	Corrected P-Value	Gene
hsa04110	Cell cycle	16	2.83E-13	MCM7, CDKN2A, TTK, CDC25C, CDC25A, CCNA2, CDC20, CCNB2, CDC45, CCNE1, CDK2, CDK1, MCM4, MCM5, MCM6, BUB1
hsa05206	MicroRNAs in cancer	10	0.001348337	PLAU, CCNE1, CDKN2A, CDCA5, STMN1, KIF23, BRCA1, CDC25C, MMP9, CDC25A
hsa05200	Pathways in cancer	10	0.01098949	CXCL8, CCNE1, STAT1, CDKN2A, CDK2, SLC2A1, CKS2, BIRC5, MMP9, CKS1B
hsa04114	Oocyte meiosis	8	7.59E-05	CDC20, CCNB2, CCNE1, CDK2, CDK1, CDC25C, BUB1, AURKA
hsa03030	DNA replication	7	8.78E-07	FEN1, RFC4, RNASEH2A, MCM7, MCM4, MCM5, MCM6
hsa04914	Progesterone-mediated oocyte maturation	7	1.56E-04	CCNA2, CCNB2, CDK2, CDK1, CDC25C, BUB1, CDC25A
hsa05161	Hepatitis B	7	0.002349213	CCNA2, CXCL8, CCNE1, STAT1, CDK2, BIRC5, MMP9
hsa05203	Viral carcinogenesis	6	0.043708607	CCNA2, CDC20, CCNE1, CDKN2A, CDK2, CDK1
hsa03460	Fanconi anemia pathway	5	0.001502456	FANCI, RMI2, FANCD2, UBE2T, BRCA1
hsa04115	p53 signaling pathway	5	0.003563288	CCNB2, CCNE1, CDKN2A, CDK2, CDK1
hsa04620	Toll-like receptor signaling pathway	5	0.0175739	CXCL10, CXCL9, CXCL8, STAT1, SPP1
hsa05222	Small cell lung cancer	4	0.045981602	CCNE1, CDK2, CKS2, CKS1B
hsa01100	Metabolic pathways	15	0.013346556	CDA, PLA2G4F, ALOX12, ALOX12B, CYP3A5, CYP2C18, TM7SF2, HAL, RDH12, ACOX2, TST, SPTLC3, MGLL, PNLIPRP3, ATP6V1C2
hsa00590	Arachidonic acid metabolism	4	0.006364075	PLA2G4F, GPX3, ALOX12B, ALOX12
hsa04726	Serotonergic synapse	4	0.031631571	PLA2G4F, ALOX12B, ALOX12, CYP2C18

### Establishment of PPI network and screening of hub genes

**Fig. 4:**
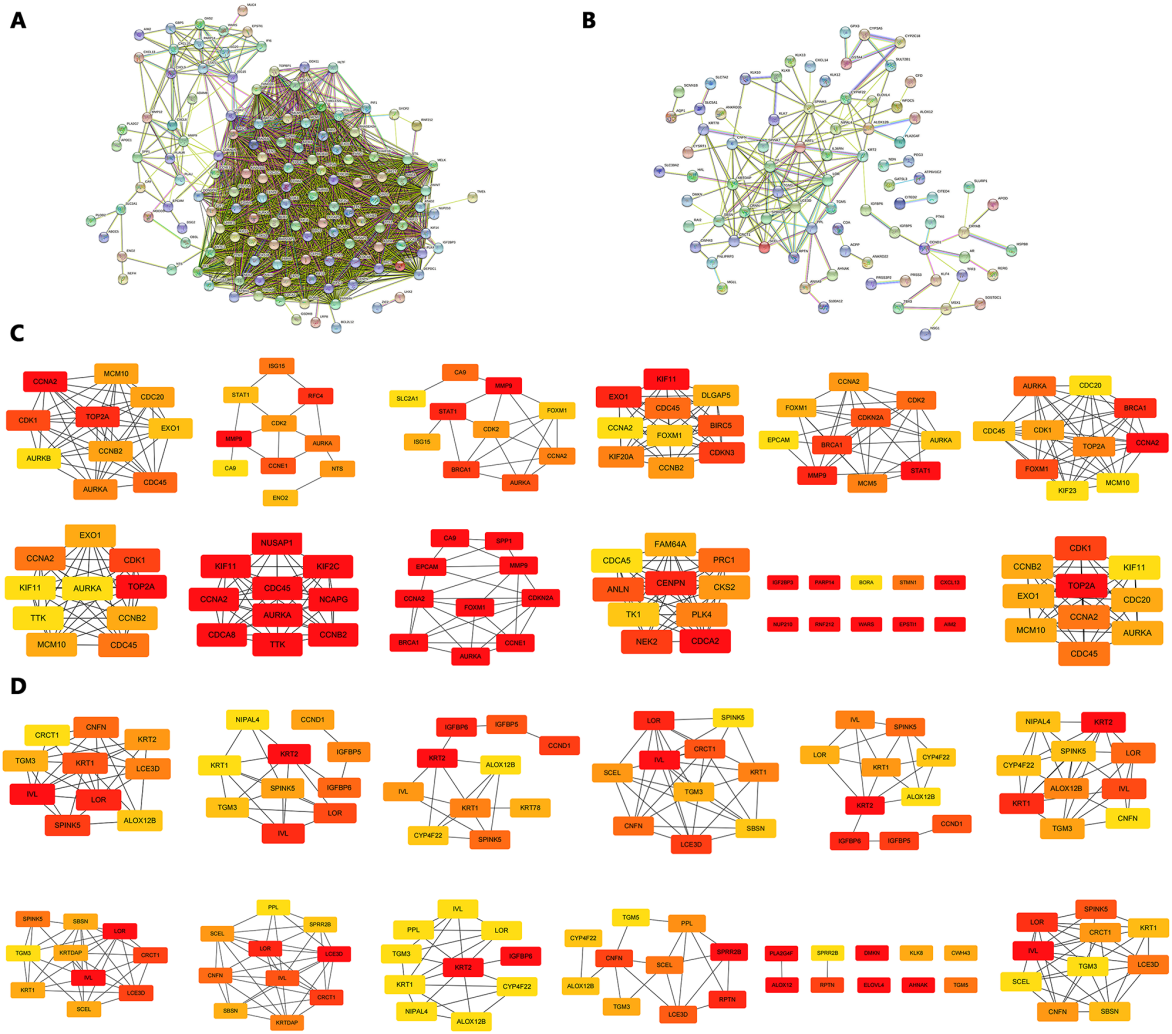
The protein-protein interaction network of DEGs and hub genes. (**A**) The PPI network constructed for up-regulated DEGs. (**B**) The PPI network constructed for down-regulated DEGs. (**C**) Top 10 genes of 12 algorithms

The 153 up-regulated genes and 144 down-regulated genes screened were analyzed using the STRING online database, and then PPI networks were constructed for these genes, as shown in Fig. 4A-B. The PPI network was further analyzed by Cytoscape software. Based on 12 algorithms (EcCentricity, DMNC, MCC, MNC, Betweenness, ClusteringCoefficient, BottleNeck, Closeness, Radiality, Stress, EPC and Degree) in cytoHubba plugin of Cytoscape, we selected the top 10 hub genes of the 12 algorithms respectively, and then obtained *AURKA* and *CCNA2* (the up-regulated genes) and *KRT1* (the down-regulated gene) with the most frequent occurrences from the above screened genes. Detailed results were shown in Table S2 and Fig. 4C-D. At the same time, *TOP2A* (the up-regulated gene) and *IV*L (the down-regulated gene) with the highest Degree score were selected as hub genes for follow-up study.

### Screenning the target genes of miR-21and miR-203

**Fig. 5:**
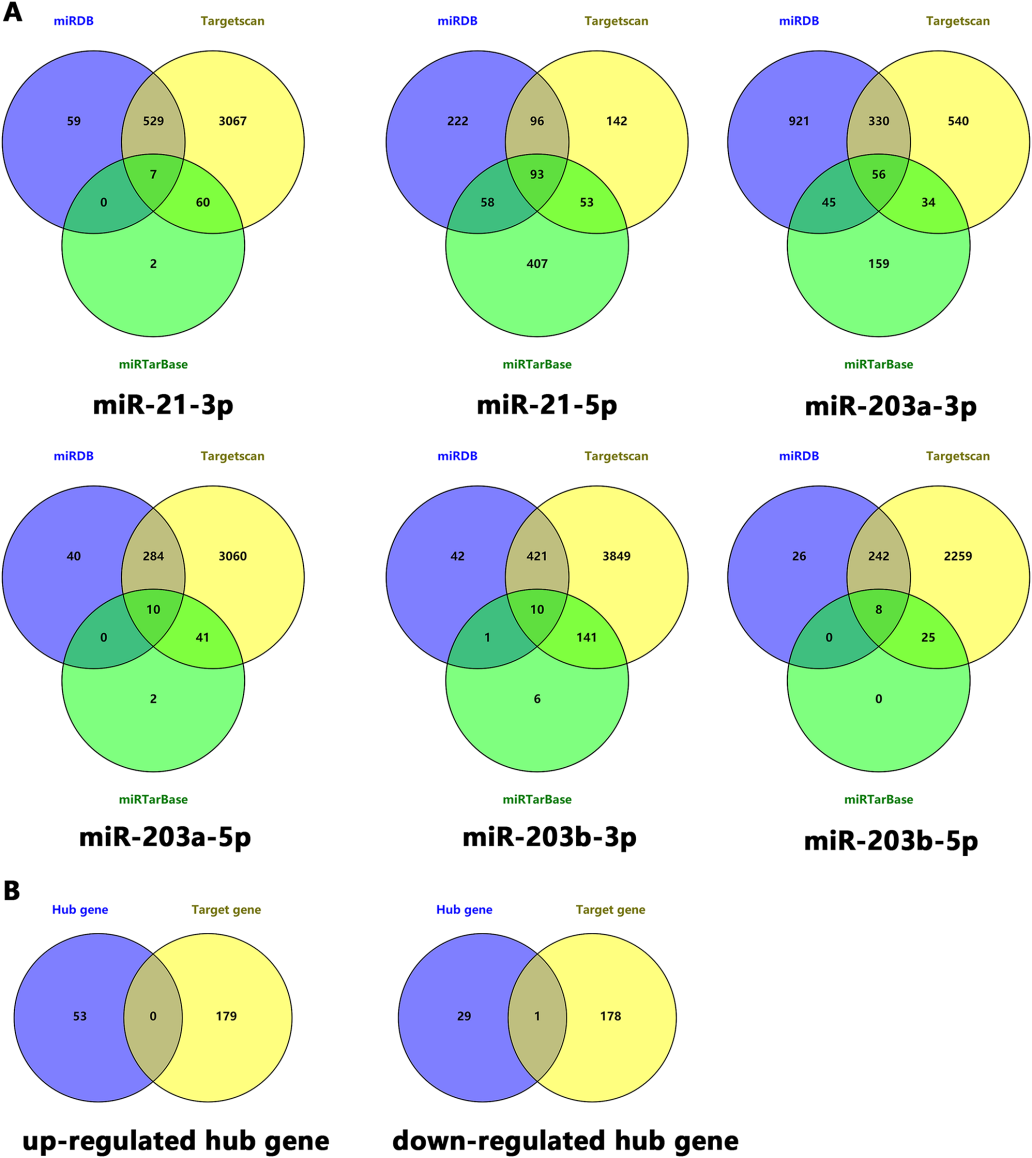
Screening hub genes by target genes of miRNA. (**A**) Screening target genes of miR-21 and miR-203 by TargetScan, miRTarBase and miRDB. (**B**) The target genes intersected with the up-regulated hub genes and down-regulated hub genes respectively

Three websites, TargetScan, miRTarBase and miRDB, were used to screen the target genes of miR-21 and miR-203, which miR-21 included miR-21-3p and miR-21 -5p, and miR-203 included miR-203a-3p, miR-203a-5p, miR-203b-3p and miR-203b-5p (the details of target genes in table S3-S4). 184 common target genes were selected by tooking the intersection of these target genes, and the results and details of these genes were shown in Fig. 5A as well as Table S5. The 184 target genes were intersected with the hub genes (53 up-regulated and 30 down-regulated, seen Table S1) screened by the above 12 algorithms to screen out IGFBP5, and the results were shown in Fig. 5B.

### Assessment of 6 hub genes for the predictive capabilities

**Fig. 6:**
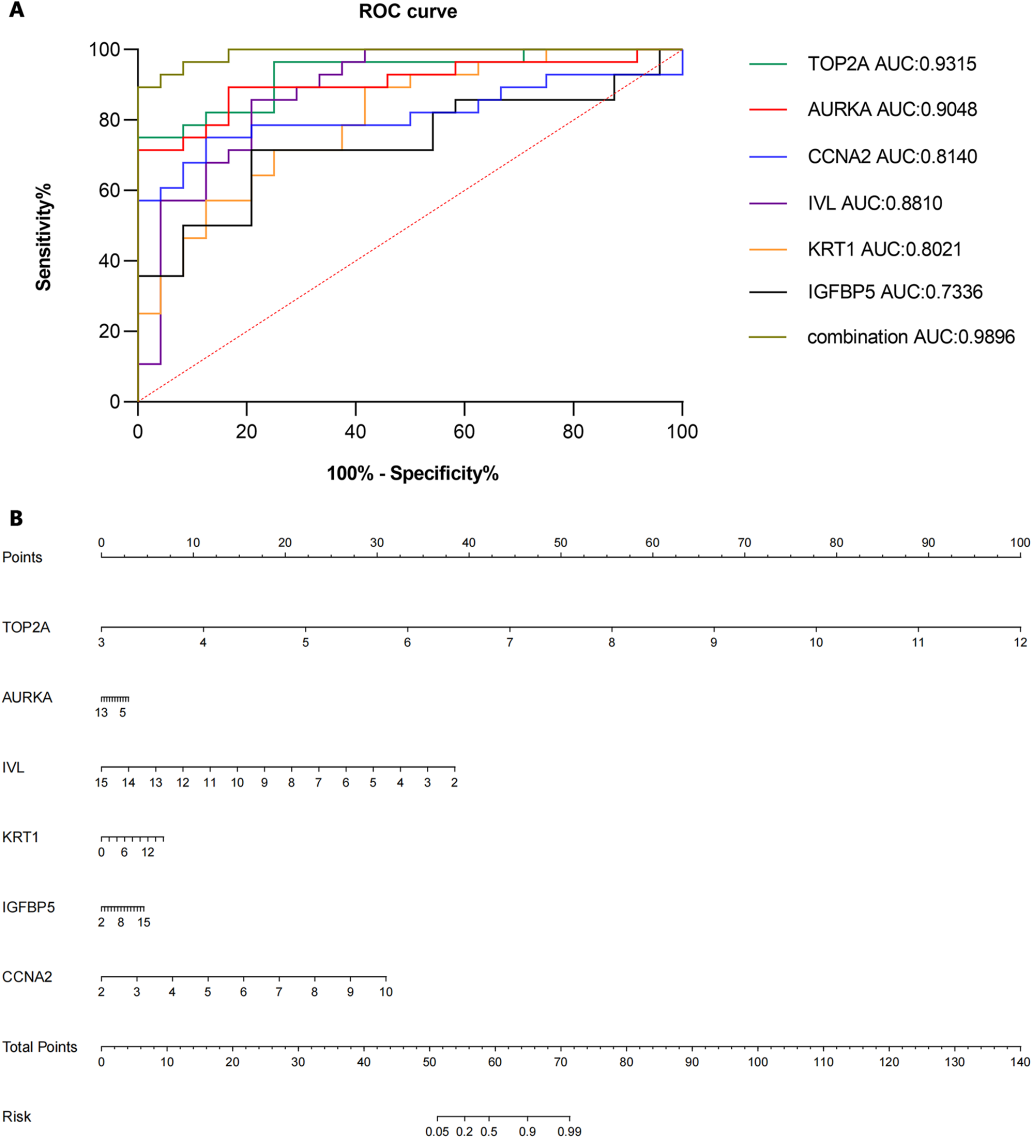
ROC curve and nomogram analysis. (**A**) ROC curve analysis of six hub genes. (**B**) nomogram analysis of six hub genes

ROC curve and nomogram analysis of the GES63514 dataset were performed to assess the predictive capabilities of the identified genes. Using the Hmisc and rms packages, we developed the nomogram model of cervical cancer based on the hub genes. As shown in Fig. 6A, in the GSE63514 dataset, the AUC values of *TOP2A*, *AURKA*, *CCNA2*, *IVL*, *KRT1*, and *IGFBP5* were 0.9315, 0.9048, 0.8140, 0.8810, 0.8021, and 0.7336, respectively. And the combined predictive value of the 6 genes was 0.9896. The results indicated that all 6 genes had predictive value in cervical cancer, and the combined predictive value of the 6 genes was more significant. As shown in Fig. 6B, the value range of these 6 genes and their contribution to the risk of cervical cancer were visualized. Based on the values of different genes in the sample, the total score can be calculated and the risk of cervical cancer can be predicted.

### Validation of hub genes

**Fig. 7:**
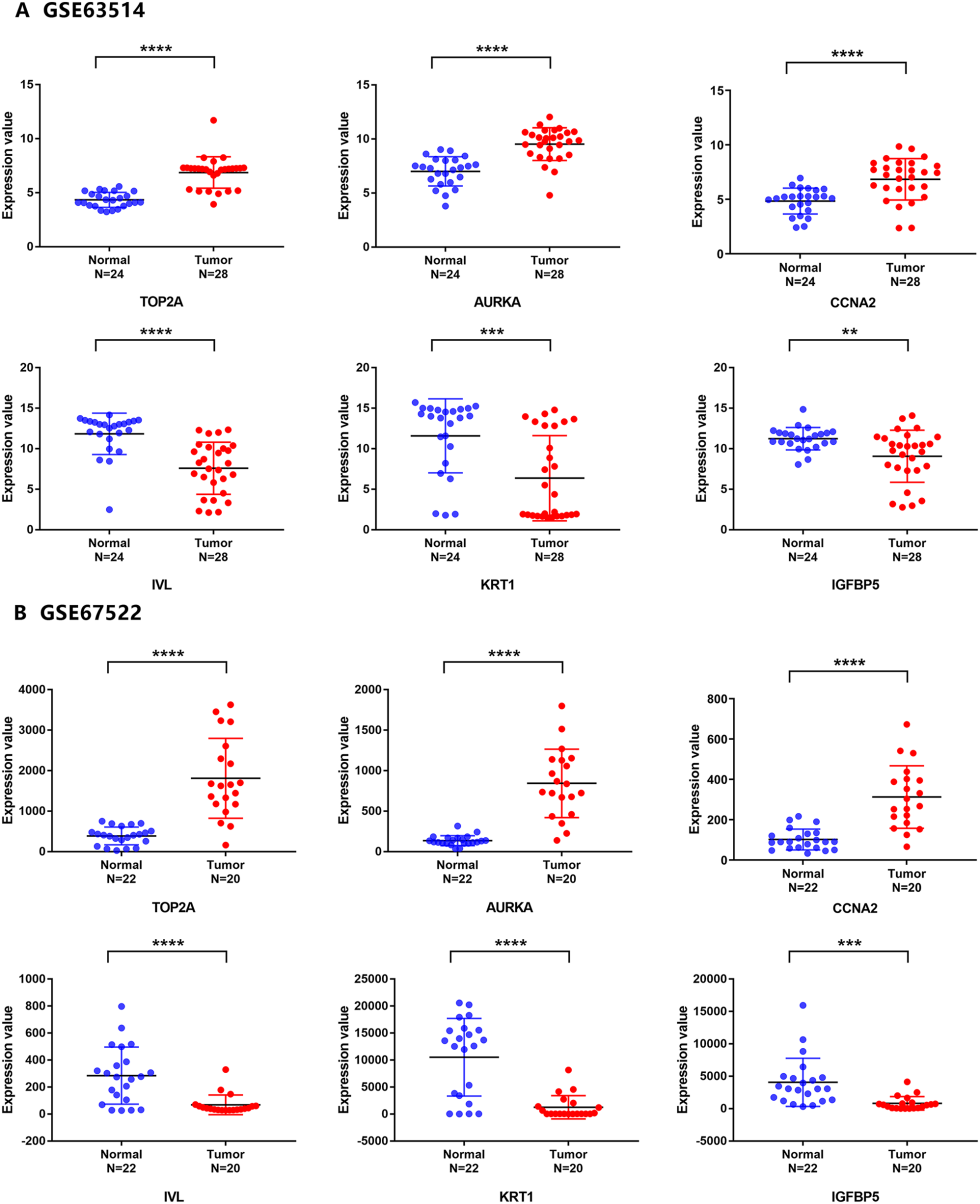
Relative expression of six hub genes in GSE63514 and GSE67522. (**A**) Relative expression of six hub genes in GSE63514. (**B**) Relative expression of six hub genes in GSE67522. **P < 0.01, ***P < 0.001, ****P < 0.0001

**Fig. 8:**
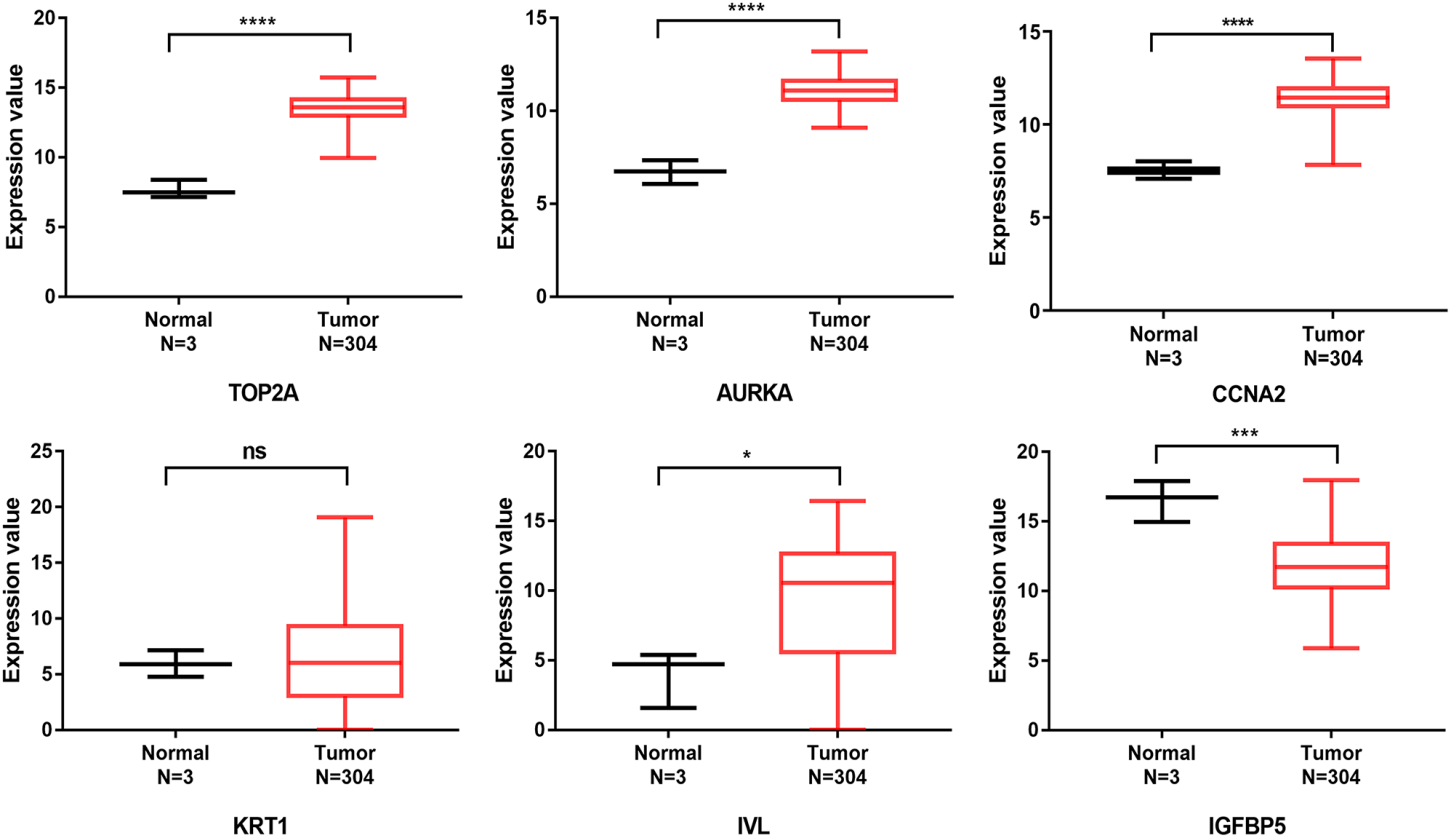
Relative expression of six hub genes in TCGA database. The above datas were analyzed from 304 cervical cancer samples and 3 normal cervix samples in TCGA database. *P < 0.05, ***P < 0.001, ****P < 0.0001

**Fig. 9:**
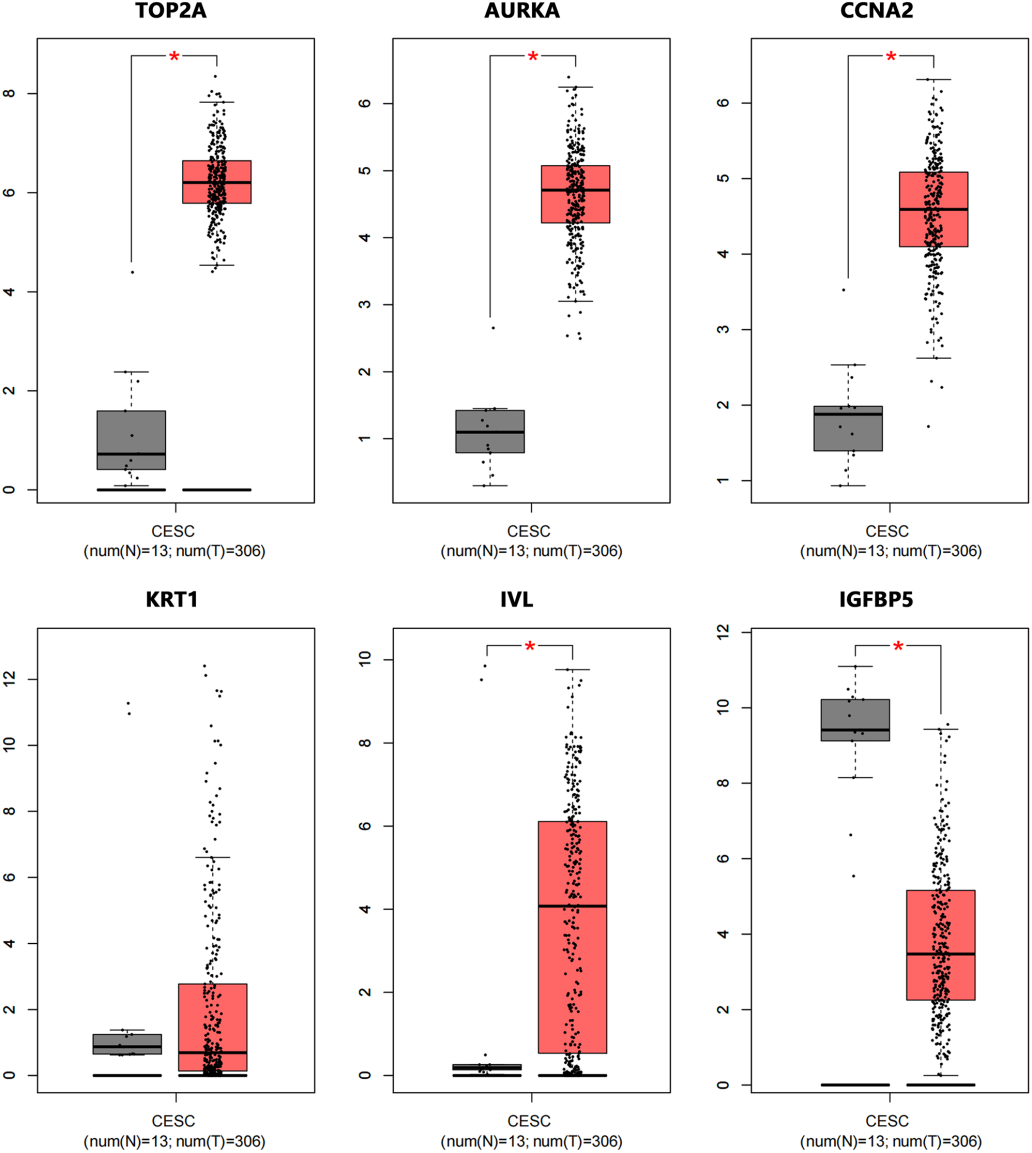
Relative expression of six hub genes in GEPIA database. The above datas were analyzed from RNA sequencing expression data of 306 cervical cancer samples and 13 normal cervix samples in GEPIA database. *P < 0.05

**Fig. 10:**
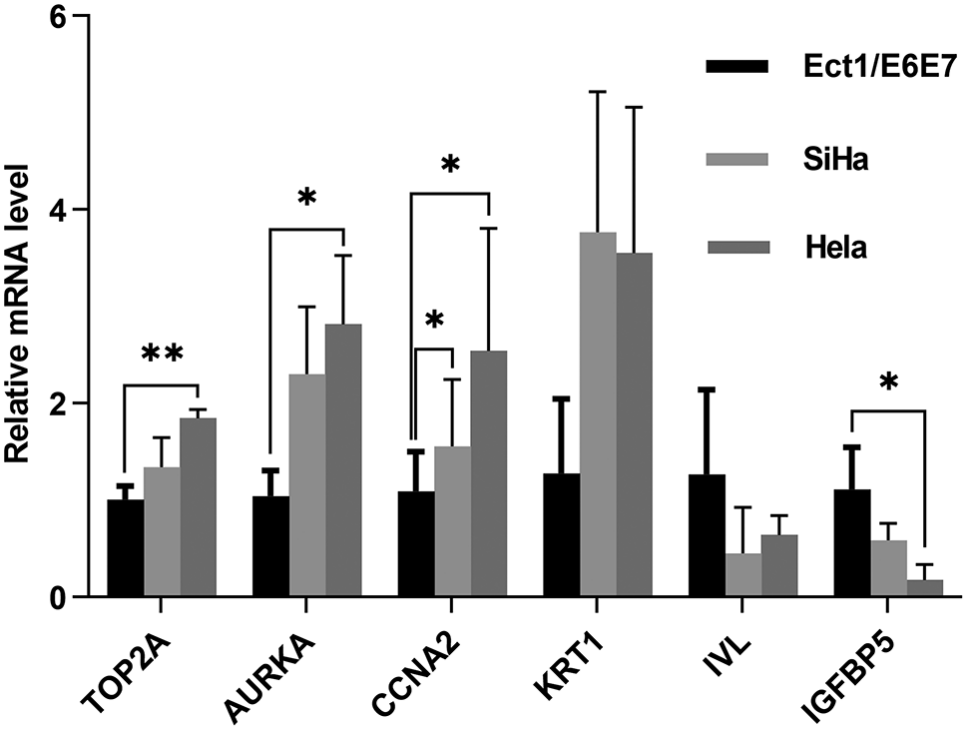
The relative mRNA level of six genes in cervical cancer cell lines. Ect1/E6E7 is human cervix immortalized squamous cell. SiHa and Hela cell are cervical cancer cell. *P < 0.05, **P < 0.01

First, we verified the six screened genes which were *TOP2A*, *AURKA*, *CCNA*2, *IV*L, *KRT1*, and *IGFBP*5, in GSE63514 and GSE67522 datasets. As shown in Fig. 7A-B, the *TOP2A*, *AURKA* and *CCNA2* in cervical cancer were significantly increased, while *IVL*, *KRT1*, and *IGFBP5* were significantly decreased, compared with normal tissues, which was consistent with the screening results. Next, we verified these 6 genes through the TCGA database.We found that *TOP2A*, *AURKA*, *CCNA2* were overexpressed and *IGFBP5* was low expression in cervical cancer, but *IVL* was overexpressed in cervical cancer, which was contrary to the result of GEO dataset in Fig. 8 and *KRT1* was no significant difference in cervical cancer tissue and normal tissue. Then, we verified the expression of these 6 genes through GEPIA (merged the normal samples of cervical tissues in the GTEx database). As shown in Fig. 9, compared with normal tissues, *TOP2A*, *AURKA*, *CCNA2* and *IVL* in cervical cancer tissues were significantly up-regulated, while *IGFBP5* was significantly down-regulated, and there was no statistical difference in the expression of *KRT1* in cervical cancer tissues and normal tissues, which were consistent with the analysis results of TCGA database. We performed staging analysis for these six genes by GEPIA. As shown in Figure S1, the cervical cancer patients with clinic Stage II, Stage III or Stage IV had a higher expression level of *AURKA*, *CCNA2*, *IVL* and *KRT1* than Stage I. Since the database analysis results were inconsistent, we detected the relative expression levels of the 6 genes in cervical cancer cell lines by qRT-PCR. As shown in Fig. 10, the expressions of *TOP2A* and *AURK*A were significantly increased in Hela cell, but the increased expressions of *TOP2A* and *AURKA* in SiHa cell were no statistical significance compared to human cervix immortalized squamous cell. And *CCNA2* were significantly increased in Hela cell and SiHa cell. The expression of *IGFBP5* was significantly decreased in Hela cell, but the decreased expression in SiHa cell was no statistical significance compared to Ect1/E6E7 cell. The expression of *KRT1* was increased in cervical cancer cell, but there was no statistical significance compared with normal cell, and, interestingly, this was contrary to the trend of *KRT1* expression in GEO datasets. The expression of *IVL* was decreased in cervical cancer cell, but there was no statistical significance, which was consistent with the expression trend in GEO datasets.

### Validation of protein

**Fig. 11:**
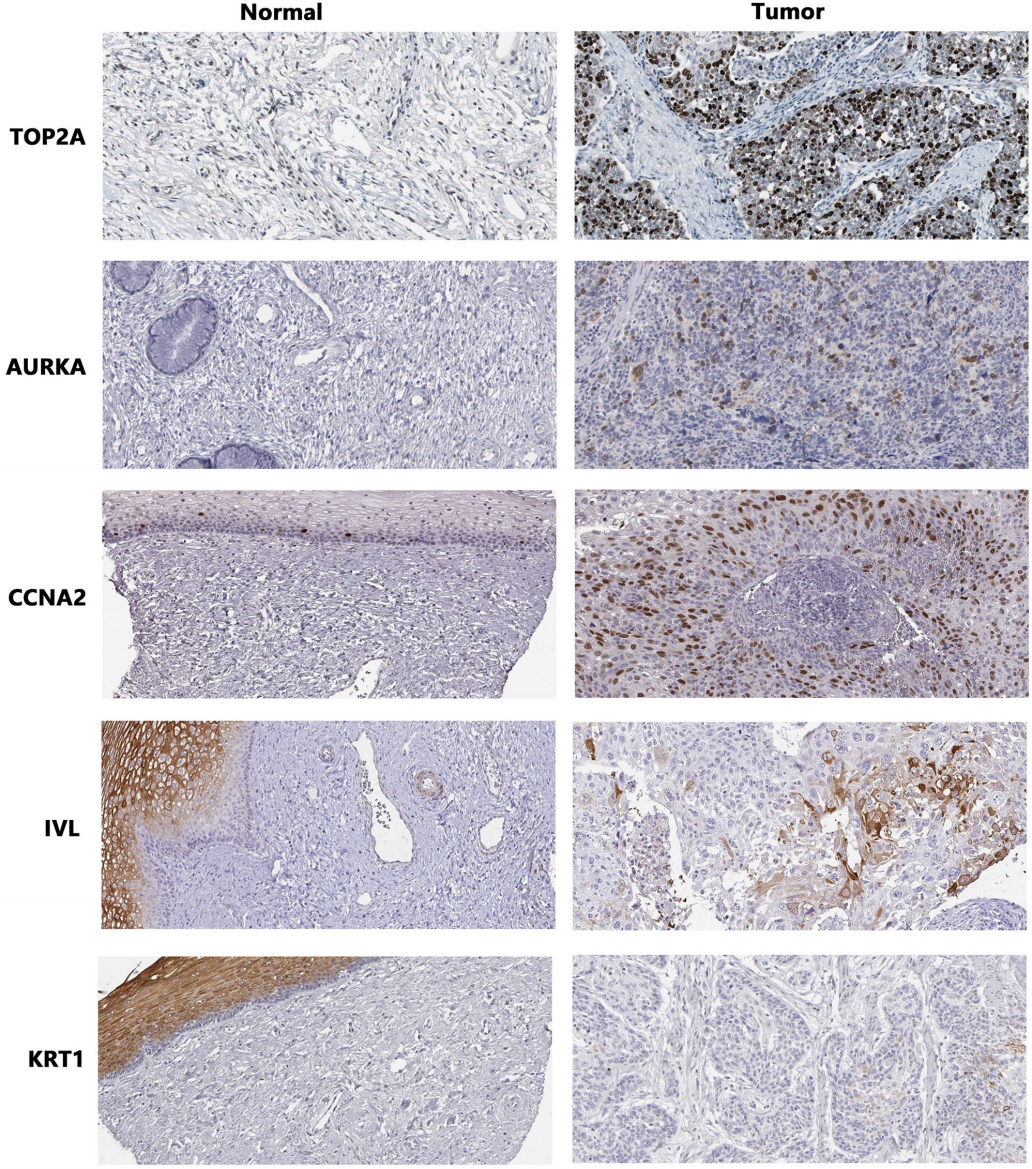
The representative immunohistochemical results of six gene in cervical cancer tissue. The positive degree was judged based on the staining intensity and percentage of stained cells

Therefore, immunohistochemical analysis of *TOP2A*,* AURKA*,* CCNA2*,* KRT1*,* IVL* and *IGFBP5* were performed through The Human Protein Atlas database and it revealed that these protein were positive in cervical cancer tissues except *IGFBP5*. The antibody HPA006458 was used to detect *TOP2A* at medium intensity with the proportion of stained cells < 25% in normal cervix tissues, while in cervical cancer tissues, it showed high intensity staining with the proportion of stained cells ranging from 25 to 75%. The antibody CAB001454 did not detect *AURKA* in normal cervix tissues, and showed moderate intensity staining in cervical cancer tissues, with the proportion of stained cells ranging from 25 to 75%. The antibody CAB000114 was used to detect *CCNA2* at medium intensity with the proportion of stained cells < 25% in normal cervix tissue, and it showed high intensity staining in cervical cancer tissue, with the proportion of stained cells from 25 to 75%. The antibody CAB002153 did not detect *KRT1* in normal cervix tissues, and it was stained with low intensity in cervical cancer tissues, and the proportion of stained cells was < 25%. The antibody HPA055211 was used to detect *IVL* at medium intensity with the proportion of stained cells < 25% in normal cervix tissues and cervical cancer tissues. Immunohistochemical results of *IGFBP5* were not included in this database. From the above results, it can be seen that the protein expression of TOP2A, AURKA and CCNA2 were significantly increased in cervical cancer tissues, as shown in Fig. 11.

### Expression of miR-21 and miR-203 in cervical cancer cell

**Fig. 12:**
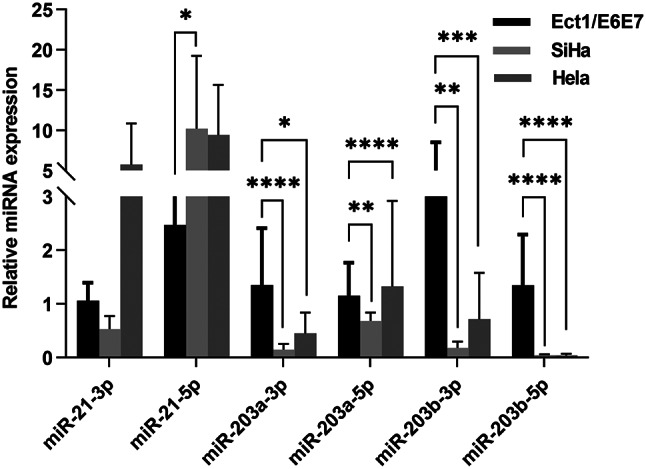
The expression level of miR-21and miR-203 in cervical cancer cell. Ect1/E6E7 is human cervix immortalized squamous cell. SiHa and Hela cell are cervical cancer cell. miR-21 includes miR-21-3p and miR-21-5p. miR-203 family consists of miR-203a-3p, miR-203a-5p, miR-203b-3p and miR-203b-5p. *P < 0.05, **P < 0.01, ***P < 0.001, ****P < 0.0001

The expression of miR-21-3p, miR-21-5p, miR-203a-3p, miR-203a-5p, miR-203b-3p and miR-203b-5p were detected in cervical cancer cells by qRT-PCR. As shown in Fig. 12, compared with human cervix immortalized squamous cell, the expression of miR-21-3p was up-regulated in Hela cell, but there was no statistical significance. MiR-21-5p was significantly up-regulated in SiHa cell compared to Ect1/E6E7 cell, while miR-203a-3p, miR-203a-5p, miR-203b-3p and miR-203b-5p were significantly down-regulated in SiHa and Hela cell.

## Discussion

GSE63514 included 24 normal and 28 cancers specimens, which were cryosecrtioned and used for laser-capture, RNA extraction, two rounds of T7-mediated amplification, and cRNA biotinylation. Bio-cRNA was hybridized to Affymetrix U133-Plus2.0 arrays, and scanned signals were processed through GC-RMA [12]. GSE67522 included 22 normal and 28 cancers specimens. Total RNA obtained from HPV negative histologically normal controls and HPV16 positive cervical cancers having either low or high HOTAIR expression levels were compared to identify transcriptome level differences [13, 14].In this study, six DEGs, *TOP2A*, *AURKA*, *CCNA2*, *KRT1*, *IVL* and *IGFBP5*, were screened out through the GSE63514 and GSE67522 datasets. *TOP2A*, *AURKA* and *CCNA2* were overexpressed in cervical cancer, while *IVL* and *IGFBP5* were low expression in cervical cancer. However, interestingly, the TCGA database and GEPIA online website analysis showed that *TOP2A*, *AURKA*, *CCNA2* and *IVL* were overexpressed and *IGFBP5* was low expression in cervical cancer, and there was no statistical difference in the expression of *KRT1* in cervical cancer tissue and normal tissue. The above inconsistent results may be due to the small number of normal tissue samples in TCGA and GEPIA database, which led to the bias in statistical analysis. Next, we extracted RNA from cultured cell to verify the expression of these 6 genes in cervical cancer cell by RT-qPCR. The results showed that the expression of *TOP2A*, *AURKA* and *CCNA2* were statistically increased in cervical cancer cell, while the expression of *IGFPB5* was statistically decreased in cervical cancer cell, which was consistent with the results of GEO dataset and TCGA database. The increased expression of *KRT1* in cervical cancer cell was not statistically significant, and interestingly, which was contrary to the expression trend of *KRT1* in GEO datasets. The expression of *IVL* was decreased in cervical cancer cell, but there was no statistical significance, which was consistent with its expression trend in GEO dataset. The *KRT1* was interesting and deserved further study. Immunohistochemical results showed that the expression of *TOP2A*,* AURKA*,* CCNA2 and KRT1* were increased in cervical cancer tissue, and the expression of *IVL* was no significant difference between cervical cancer tissue and normal cervix tissue.

*TOP2A* (DNA topoisomerase II alpha) encodes a DNA topoisomerase, an enzyme that controls and alters the topologic states of DNA during transcription. This nuclear enzyme is involved in processes such as chromosome condensation, chromatid separation, and the relief of torsional stress that occurs during DNA transcription and replication. The gene encoding this enzyme functions as the target for several anticancer agents and a variety of mutations in this gene have been associated with the development of drug resistance [15]. It was reported that the expression of *TOP2A* was up-regulated in cervical cancer and it promoted cell migration, invasion and epithelial-mesenchymal transition in cervical cancer by activating the PI3K/AKT signaling [16].

The protein encoded by *AURKA* (aurora kinase A) is a cell cycle-regulated kinase that appears to be involved in microtubule formation and/or stabilization at the spindle pole during chromosome segregation. The encoded protein is found at the centrosome in interphase cells and at the spindle poles in mitosis. This gene may play a role in tumor development and progression [17]. *AURKA* was overexpressed and associated with lymph-node metastasis in cervical cancer patients [18].

The protein, cyclin A2, encoded by *CCNA2* belongs to the highly conserved cyclin family. This protein binds and activates cyclin-dependent kinase 2 and thus promotes transition through G1/S and G2/M [19].It was reported that *CCNA2* was up-regulated and the high *CCNA2* expression promoted cell cycle progression in cervical cancer [20].

The protein encoded by *KRT1* (keratin 1) is a member of the keratin gene family, which is located in the epithelial prickle and granular cell layer. *KRT1* has been proved to regulate kinase activity and participate in angiogenesis, fibrinolysis and oxidative stress [21]. so far, there are few studies on *KRT1* in cervical cancer.

Involucrin encoded by *IVL* is a component of the keratinocyte crosslinked envelope, which is found in the cytoplasm and crosslinked to membrane proteins by transglutaminase [22]. *IVL* was significantly downregulated in cervical intraepithelial neoplasia and ultimately squamous cell carcinoma [23].

Insulin like growth factor binding protein 5 encoded by *IGFBP5 is* involved in several processes, including cellular response to cAMP and regulation of smooth muscle cell migration as well as regulation of smooth muscle cell proliferation [24, 25]. *IGFBP5* was downregulated in cervical squamous cell carcinomas tissues samples [26]. IGFBP5 expression was up-regulated in response to progression of CIN and down-regulated in invasive cervical carcinoma [27].

miRNA are short (20–24 nt) non-coding RNAs that are involved in post-transcriptional regulation of gene expression in multicellular organisms by affecting both the stability and translation of mRNAs. miRNA can be divided into three different forms, which include primary (pri‑) miRNA, precursor (pre‑) miRNA and mature miRNA. The mature miRNA called miRNA-3p and miRNA-5p are derived from the 3’ or 5’ arm of their pre-miRNA, respectively. Therefore, all pre‑miRNAs can produce both types of mature miRNA [28, 29]. In this study, miR-21 (up-regulated) and miR-203 (down-regulated) were screened out through the GSE30656 dataset, which analysed 10 squamous cell carcinomas of the cervix, 9 adenocarcinomas of the cervix and 10 cervical squamous epithelial samples with normal histology using single channel (Cy3) miRNA microarrays from Agilent [30]. MiR-21 includes miR-21-3p and miR-21 -5p, and miR-203 family includes miR-203a-3p、miR-203a-5p、miR-203b-3p and miR-203b-5p. The miR-21-3p and miR-21-5p were up-regulated in cervical cancer cells, while miR-203a-3p, miR-203a-5p, miR-203b-3p and miR-203b-5p were down-regulated in cervical cancer cells by RT-qPCR, which was consistent with the analysis results of GSE30656 dataset. MiR-21 acts as an oncogene in cancer by regulating many pathways involved in tumor development. MiR-21 was up-regulated and regulated multiple signaling pathways in cervical cancer, including TNF-α/caspase-3/caspase-8, PI3K/AKT/mTOR, and RAS/MEK/ERK pathways [31]. MiR-203 in the cervical cancer group was significantly lower than control group [32]. And miR-203 was involved in cell cycle regulation and suppressed cervical cancer cell migration and invasion [33].

The advantage of this study was that it innovatively combined mRNA and miRNA datasets to screen hub genes, and the 6 selected hub genes were verified by RT-qPCR and immunohistochemistry. It was found that TOP2A, AURKA, CCNA2 and IGFBP5 could be all potential tumor markers for cervical cancer. It was worth noting that IGFBP5 was neither the top 10 DEGs screened by the mRNA datasets nor the top 10 target genes screened by the miRNA dataset, but it was the only gene that appeared in both screening pathways at the same time, so it was selected as a hub gene. This provided a new idea for mining hub genes.

However, Our study also had some limitations. For example, all the data analyzed in this study were from bioinformatics databases. And this study only screened out hub genes related to cervical cancer tumors, but their internal influencing mechanisms and interconnections were not clear. It is required further research in cervical cancer by clinical tissues and cervical cancer cell lines in vivo and in vitro experiments.

## Conclusions

In short, through bioinformatics analysis, as well as qRT-PCR and immunohistochemistry, this study found that TOP2A, AURKA, CCNA2 were overexpressed and IGFBP5 was low expression in cervical cancer, which might be potential tumor markers and further research is needed to confirm their clinical value.

### Electronic supplementary material

Below is the link to the electronic supplementary material.

Supplementary Material 1

Supplementary Material 2

Supplementary Material 3

Supplementary Material 4

Supplementary Material 5

Supplementary Material 6

## Data Availability

The datasets that support the findings of this study are available in the GEO database (https://www.ncbi.nlm.nih.gov/geo/).

## Abbreviations

DEGs: Differentially expressed genes
CC: Cervical cancer
SCC: Squamous cell carcinoma
ADC: Adenocarcinoma
ADSC: Adenosquamous carcinoma
GO: Gene Ontology
KEGG: Kyoto Encyclopedia of Genes and Genomes
PPI: Protein-protein interaction
DEMs: Differentially expressed miRNAs
TCGA: The Cancer Genome Atlas
GEPIA: Gene Expression Profile Interaction Analysis
ROC: Receiver Operating Characteristic
AUC: Area Under the Curve
cDNA: Complementary DNA
MF: Molecular Function
CC: Cellular Component
BP: Biological Process
TOP2A: DNA Topoisomerase II Alpha
AURKA: Aurora Kinase A
KRT1: Keratin 1
