# Supplementary figures and images for: Identification of key genes associated with cervical cancer based on bioinformatics analysis
Source: BMC Cancer. 2024 Jul 25;24:897. doi: 10.1186/s12885-024-12658-z (PMC11282596; doi:10.1186/s12885-024-12658-z)

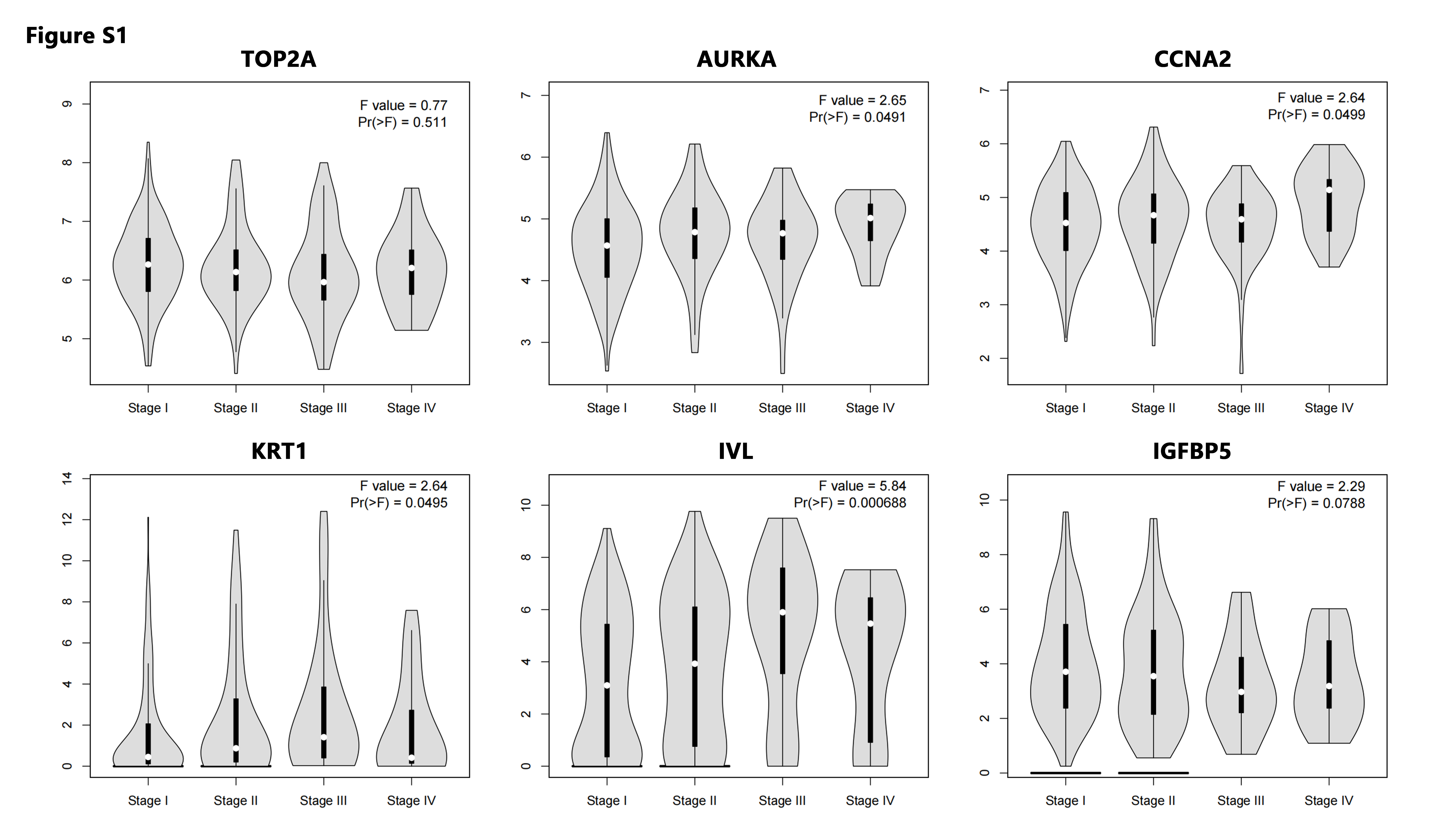

Supplement: Supplementary file 1 — Supplementary Material 1 [file 12885_2024_12658_MOESM1_ESM.tif]
